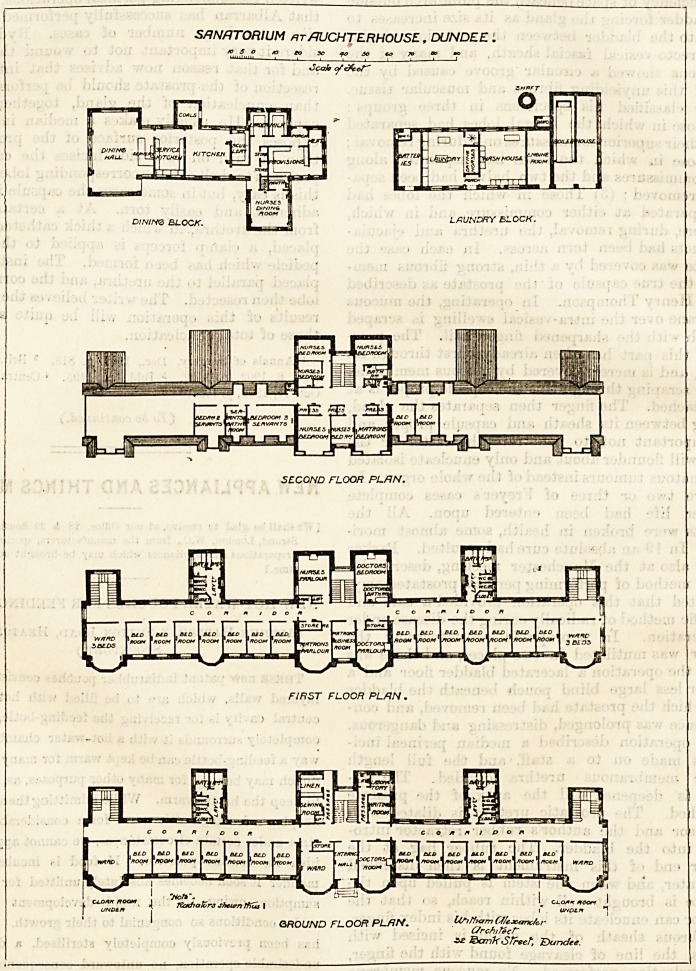# Sanatorium for Consumptives at Auchterhouse, Dundee

**Published:** 1903-03-14

**Authors:** 


### HOSPITAL ADMINISTRATION.

### CONSTRUCTION AND ECONOMICS.

### SANATORIUM FOR CONSUMPTIVES AT AUCHTERHOUSE, DUNDEE.

This sanatorium has been built about eight miles from Dundee, and was opened last autumn by the Countess of Airlie. It occupies a fine site on the south slope of the Sidlaw Hills; is about 800 feet above the sea level, faces due south, and is sheltered from the north and east by wooded hills which rise to a height of 1,400 feet. Being open to the south and to the west, the sanatorium commands extensive views of the river Tay, and of the hills of Fife and Lomond. In the background to the west are the Perthshire hills, and 40 miles off Schiehalion is seen towering over the nearer hills.

The main building is of the Lineal type, the general conception being good, and the elevation decidedly pleasing. It consists of a centre and two wings. The former contains the entrance-hall, medical officer's room, laboratory, waiting-room, sewing-room, and staircase. There is also a ward apparently for two beds. Each wing contains seven separate bedrooms and a ward for three beds, the latter being placed at the extreme end of the wing. A corridor runs at the north side of the building, and projecting northwards from this are the sanitary blocks and the staircases. As regards the separate bedrooms they are correctly planned, and if the doors be left constantly open, or if sufficient open space be left over the doors, there ought to be plenty of cross ventilation in these rooms save, perhaps, in those opposite which the sanitary blocks are placed. As regards the dormitory wards they cannot be described as altogether satisfactory. Only one end and one side are free to the outside air; but it will be seen that the south end has a large bay window, and that the side has another, but smaller, window, and these somewhat reduce the evil. This part of the sanatorium would have been immensely improved if the staircase had been placed where the ward is, and the ward placed with its long diameter facing south and north, and with windows on both of these aspects. By making the ward a few feet longer it might have contained four beds, and the present two-bedded wards should have been given up to some administrative purpose. The sanatory blocks are sufficient in size, and they are fairly well arranged, but they ought to have been entirely cut off from the corridor by a short cross-ventilated passage, and they should have been placed where the staircase now is. For some reason sitting-rooms have been omitted from the plan.

The first floor is practically a replica of the ground floor. The second floor contains the bedrooms for the staff. The block containing the dining-hall, kitchen and offices is well planned, and some advantages accrue from its being placed apart from the main building; among others, the smell of cooking does not invade the sanatorium. The laundry block is conveniently arranged. The sanatorium is designed for 42 patients. Of these, 28 have separate bedrooms.

There is an electric-light installation, and the building is warmed partly by open fireplaces and partly by radiators on the low-pressure system. As points of some importance, it may be mentioned that all the corners have been rounded off, and there are no cornices and no elaborate mouldings anywhere to act as dust traps. The furniture is of simple description, and it is made to run on castors so that every article may be easily moved.

The sanatorium owes its existence to the munificence of ex-provost Moncur, who contributed £15,000. Lord Airlie presented the site. Mr. Cox and Mr. Fleming each gave one thousand guineas, and an anonymous donor contributes £500 a year for five years. Here are examples which we should like to see widely followed on this side of the Tweed. The total cost of the sanatorium was £20,000, being a little over £475 a bed. This seems a large sum per bed. The architect was Mr. William Alexander of Dundee.

## Figures and Tables

**Figure f1:**